# Directional motion of a self-steering active intruder in a dense crowd of cognitive active agents

**DOI:** 10.1038/s41598-026-52749-4

**Published:** 2026-06-15

**Authors:** Vikas Kumar Kushwaha, Priyanka Iyer, Sunil P. Singh, Gerhard Gompper

**Affiliations:** 1 Department of Physics, Indian Institute of Science Education and Research Bhopal, Bhopal, India; 2 Theoretical Physics of Living Matter, Institute for Advanced Simulation, Forschungszentrum Jülich, 52425 Jülich, Germany

**Keywords:** Engineering, Mathematics and computing, Physics

## Abstract

The fast and efficient directed motion of particles through crowded environments is challenging problem. In this work, the surface-bound motion of an intruder in a crowd of identical active agents is studied by overdamped Langevin dynamics simulations. Both intruder and agents are modeled as intelligent active Brownian particles (iABPs) with visual perception and directional steering to avoid collisions - which implies non-reciprocal interactions between all particles. The reorientation of intruder and agents is limited by their maximal maneuverability, which controls the ability of an iABP to adjust its velocity direction. The simulation results show that the intruder’s attempt to increase directional speed by steering around agents fails; in fact, this even reduces the directional speed. In contrast, the intruder has to be perceived by the agents so that they can move out of the way in time. The intruder speed and transverse diffusivity are determined as functions of several key control parameters, like maneuverability, vision angle, and agent density. Here, an important parameter is the uniformity of the agent distribution. It is shown that the agent’s self-steering to avoid collision enhances hyperuniformity (class III), which facilitates an easier directional navigation of the intruder. Results are relevant, inter alia, for the motion of emergency personnel in semi-dense human crowds.

## Introduction

The directed motion of self-propelled particles through environments containing a maze of obstacles is an important problem which appears in nature in many guises, and in a broad range of length scales. Three categories of obstacles can be clearly identified: (i) Static arrays of solid obstacles. This occurs, for example, for large animals in a forest trying to escape from a predator, but also for bacteria moving in porous media^1^ and for self-propelled colloids in obstacle arrays^2^. (ii) Mobile and deformable obstacles. Examples include trypanosome parasites swimming in blood (a dense suspension of red blood cells)^3^, leukocytes chasing bacteria in blood^4^, bacteria^5, 6^, and self-propelled colloids^7, 8, 9^ swimming in polymer suspensions, and colloidal microswimmers in dense colloid dispersions^10^. (iii) Crowds of cognitive and reactive self-propelling agents. Such environments are encountered, for example, in crowds of pedestrians^11, 12^, when paramedics try to quickly reach an injured person in an emergency^13^.

The emergent dynamics of self-steering particles in complex environments is a general feature seen in a large variety of active systems capable of “cognition”. These typically involve mobile agents that steer based on input from local perception, often leading to self-organization such as the formation of flocks, swarms, and herds in animal groups, or lanes in bidirectional pedestrian flows. This implies that many aspects of this dynamics can generically be understood as the behavior of interacting self-propelled entities, which places it in the realm of the large field of “active matter”^14, 15, 16^, encompassing systems from cell suspensions and self-propelling colloids to schools of fish and flocks of birds^17, 18, 19, 20, 21, 22^. In this context, the active Brownian particle (ABP) model has been used extensively to understand many intriguing aspects of non-equilibrium physics. Moreover, when equipped with directional environment sensing and self-steering, ensembles of “intelligent” ABP systems (iABPs) show a rich variety of collective phenomena such as milling, single-file motion, flocking, worm-like swarms, and polar or nematic ordering^23, 24, 25, 26, 27^.

Across both biological and artificial systems, directional sensing and goal-oriented motion ultimately emerge from the interplay between environmental cues, internal decision-making, and active reorientation, providing a unifying framework for understanding navigation in complex environments. Understanding the physical mechanisms underlying active particle navigation in dense complex environments is vital, as efficient navigation in such media is central to many biological and engineered systems. Such insight underpins applications ranging from targeted drug delivery and physiological transport processes to the design of micro-machines and robophysical technologies capable of reliable operation in real-world, crowded environments^20, 28, 29^.

A particularly intriguing question is the self-organization in systems, in which an active particle moves in a medium, where both the particle and the medium are cognitive and react to the presence and activity of each other. In such a case, the dynamics of the active particles and the properties of the environment are strongly coupled with each other and affect their mutual motion^29^. The motion of a single active particle through a dense and dynamically fluctuating environment, where frequent collisions, steric interactions, and collective rearrangements disrupt persistent motion and hinder the particle’s ability to maintain a directed motion. These conditions are remarkably different from those in dilute media, where directed motion is typically easier to maintain^30, 31^. A more familiar example of this challenge is the navigation of pedestrians or micro-robots moving within dense crowds of other walkers or robots. These agents rely heavily on visual input and onboard sensing, using this information to steer, avoid collisions, and efficiently reach their targets. Experimental investigations in pedestrian and granular-matter systems have shown that the presence of an intruder is associated with the formation of a cavity trailing behind it, accompanied by lateral flow of the surrounding medium^11, 32^. Despite these similarities, unlike granular media (which have a mechanical response), pedestrians respond differently due to their perception-dependent movement, highlighting the strong influence of cognition on the dynamics of both the intruder and the bath^33^.

It is worth mentioning that directed motion has also been observed for passive particles in active environments, where it arises from activity-induced pressure gradients^34^. However, the underlying mechanisms in these two cases are fundamentally different. For passive particles, motion is controlled by externally applied (e.g., light-induced) activity gradients, whereas the motion of an active intruder is intrinsically controlled by its self-steering maneuverability. This highlights the fundamental distinction between environment-driven and self-driven control mechanisms.

In this study, we are mainly interested in elucidating the directed motion of an “intruder” - an active, self-propelled particle with visual perception and avoidance steering - in an array or crowd of “agents” - which can be passive particles, active with constant speed and rotational diffusion of the propulsion direction, or cognitive with self-steering to avoid other agents as well as the intruder. We consider two kinds of maneuverability: vision-based avoidance and goal-oriented steering. The vision-based maneuverability enables a particle to detect the position of instantaneous neighbors within their vision cone (VC) and maneuver to avoid conflicts. The intruder’s ability to move toward the goal is affected by the motion of nearby agents. A central question addressed here is which strategies enable persistent goal-oriented motion in dense crowds while simultaneously minimizing collisions - relevant, for example, to the motion of ambulances in traffic or toward a patient in a crowd.

The article is organized as follows. In Section Simulation model, we present the coarse-grained model for intelligent active Brownian particles (iABPs) and describe the realization of various maneuvering strategies. In Section Directed speed of the intruder, we examine the effects of different maneuverabilities, as well as the role of agent density and vision angle, on the intruder’s directed speed. In Section Intruder’s transverse diffusivity, we analyze the transverse diffusivity. The hyperuniformity of the agent crowd is characterized, and the importance of this quantity for the directional motion of the intruder is discussed in Section The agent crowd: minimal agent-agent distance, hyperuniformity, and void size around intruder. Finally, summary and conclusions are presented in Section Summary, discussion, and conclusions.

**Fig. 1:**
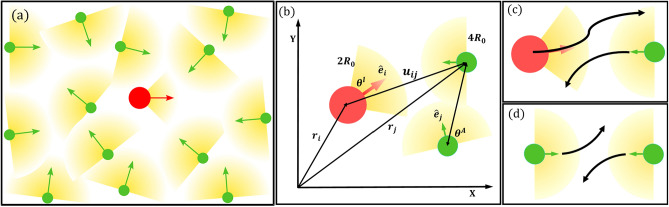
(**a**) A schematic of the system of intelligent active Brownian particles (iABPs) illustrating two kinds of particles: intruder (red) and agents (green). (**b**) Schematic representation of the vision cone of the intruder and agents with orientations ${\textbf {e}}_i { \& } {\textbf {e}}_j$, and vision angles $\theta ^I { \& } \theta ^A$. The interparticle distance between intruder-to-agents $d_1$ and agents-to-agents is $d_2$. (**c**) The schematic illustrates trajectories of the intruder and agents: the intruder shows the effect of vision and directed maneuverabilities ($M_i^v { \& } M_i^d$) by collision avoidance and subsequently reorients its heading toward the goal direction (x-axis), whereas agents only avoid the intruder. (**d**) Agents’ trajectories are demonstrating the effect of vision maneuverability $M_i^v$ by avoiding each other.

## Simulation model

The intruder and agents are modeled as *intelligent* active Brownian particles (iABPs) in a planar geometry, as shown in Fig. 1a. Here, the system consists of one intruder and $N-1$ agents.

The dynamics of a particle *i* $(i = 1,....,N)$ at position ${\textbf {r}}_i(t)$ at time *t* are governed by the equation of motion^35, 36^,

1 $$\begin{aligned} m \ddot{{\textbf {r}}}_i = - \gamma \dot{{\textbf {r}}}_i + f_{\text {a}} {\textbf {e}}_i + {\textbf {F}}_{i}. \end{aligned}$$

where *m* is the particle mass, $\gamma$ the translational friction coefficient, and $f_a$ is the magnitude of the propulsion force, which is acting along the instantaneous particle orientation vector ${\textbf {e}}_i$. The force ${\textbf {F}}_i = - \sum _{j \ne i} \partial U({\textbf {r}}_{ij})/\partial {\textbf {r}}_{ij}$ describes the (short-range) repulsive interaction with neighboring particles, which is modeled by the Weeks-Chandler-Andersen (WCA) potential

2 $$\begin{aligned} U(r_{ij}) = {\left\{ \begin{array}{ll} 4\epsilon \left[ \left( \dfrac{\sigma }{r_{ij}} \right) ^{12} - \left( \dfrac{\sigma }{r_{ij}} \right) ^6 + \dfrac{1}{4} \right] , & \text {if } r_{ij} \le 2^{1/6}\sigma \\ 0, & \text {otherwise}. \end{array}\right. } \end{aligned}$$

Here, ${{\textbf {r}}}_{ij}={\textbf {r}}_i-{\textbf {r}}_j$ with $r_{ij}=|{\textbf {r}}_{ij}|$ is the distance between iABPs *i* and *j*, $\sigma$ is the particle diameter, and $\epsilon$ is the strength of the interaction. This potential describes the excluded volume interaction between the iABPs, which prevents the close approach and contact of particles.

The cognitive properties of iABPs enable them to sense the location of other particles, and to respond by adjusting their propulsion direction in order to avoid collision. As shown in Fig. 1, particle *i* can reorient its self-propulsion direction ${\textbf {e}}_i$ through vision-controlled self-steering mechanism, which is modelled by a steering torque ${\textbf {M}}_i^v$. In addition, the intruder has the goal to move through the sea of agents with a goal direction ${\textbf {e}}_g$, which is captured by a torque ${\textbf {M}}_i^d$. Thus, the equation of motion for the particle orientation ${\textbf {e}}_i$ is

3 $$\begin{aligned} \dot{{\textbf {e}}_i} = {\textbf {M}}_i^v + {\textbf {M}}_i^d + \mathbf {\Lambda } \times {\textbf {e}}_i \end{aligned}$$

where the contribution ${\textbf {M}}_i^d$ is only present for the intruder. The stochastic term $\mathbf {\Lambda }$ denotes the Gaussian and Markovian stochastic processes, with $\langle \Lambda _{i}(t)\rangle =0$ and $\langle \Lambda _{i}(t)\Lambda _{j}(t')\rangle = 2 (d-1) D_R \delta _{ij}\delta (t-t')$, where *d* is the spatial dimension and $D_R$ refers to the rotational diffusion coefficient.

Following Ref.^35^, we employ the vision-induced avoidance torque

4 $$\begin{aligned} {\textbf {M}}_i^v = - \frac{\Omega _v}{N_{c,i}} \sum _{j \in VC} e^{-r_{ij}/R_0} \, [{\textbf {e}}_i \times ({\textbf {u}}_{ij} \times {\textbf {e}}_i)], \end{aligned}$$

where ${\textbf {u}}_{ij} = ({\textbf {r}}_i-{\textbf {r}}_j)/|{\textbf {r}}_i-{\textbf {r}}_j|$ is the directional unit vector toward the neighboring particles in the vision cone *VC*, which is defined as a sector with opening angle $2\theta$ - where $\theta$ is the vision angle - and vision range $R_v$. The exponential decay length $R_0 < R_v$ in Eq. (4) mimics the partial blocking of view by close-by neighbors on particles further away^35^. In two dimensions, the condition for particle *j* to lie inside the vision cone of particle is *j* is $\textbf{u}_{ij} \cdot \textbf{e}_i \ge \cos (\theta )$. The coefficient $\Omega _v$, which determines the maximal possible steering torque, is denoted “visual maneuverability”, as it controls the ability of an iABP to adjust its velocity direction. The normalization factor $N_{c,i}$ in Eq. (4) is the effective number of particles inside the vision cone,

5 $$\begin{aligned} N_{c,i} = \sum _{j \in VC} e^{-r_{ij}/R_0}. \end{aligned}$$

Note that in the case of only a single particle in the vision cone, the exponential-decay factor cancels out. As in Ref.^36^, the goal-fixation related torque of the intruder is defined as

6 $$\begin{aligned} {\textbf {M}}_i^d = \Omega _d \ [{\textbf {e}}_i \times ({\textbf{e}}_g \times {\textbf {e}}_i)] \end{aligned}$$

where ${\textbf{e}}_g$ is the goal direction and $\Omega _d$ is the corresponding maneuverability.

Note that particle interactions in the system with visual perception considered here are non-additive and non-reciprocal. In fact, there are several sources of non-reciprocity in the intruder-agent system^37^. Clearly, for a vision cone with a finite open angle, one particle may see and react to the other, but not vice versa. However, the interactions described by Eqs. (3), (4), and (6) are already non-reciprocal for particles with panoramic view, because a particle reacts differently to another in front and behind. Furthermore, intruder and agents react differently to the presence of the other.

In polar coordinates, the orientation vector of particle *i* is ${\textbf {e}}_i = (\cos (\phi _i), \sin (\phi _i))^T$, and the neighbor direction vector is ${\textbf {u}}_{ij} = (\cos (\psi _{ij}), \sin (\psi _{ij}))^T$. Here, $\phi _i$ is the position angle of the iABPs and $\psi _{ij}$ the angle by which particle *j* is seen from particle *i*, i.e., the polar angle of the distance unit vector ${\textbf {u}}_{ij}$, see Fig. 1b.

The system under consideration comprises two types of particles: an intruder and agents. Both particle types share identical physical and behavioral properties, except for their maneuvering strategies. Agents employ vision-induced avoidance maneuverability determined solely by ${\textbf {M}}_i^v$, whereas the intruder has to find a compromise between avoiding agents and reaching the goal, so that both vision-induced avoidance maneuverability and goal-directed maneuverability ${\textbf {M}}_i^v + {\textbf {M}}_i^d$ are present.

From Eq. (3), the equation of motion for the orientation angle $\psi _i$ for intruder (*I*) and agents (*A*) are obtained, respectively, as

7 $$\begin{aligned} {\dot{\phi }}_i^I&= - \frac{\Omega _v^I}{N_{c,i}} \sum _{j \in VC} e^{-r_{ij}/R_0} \sin ( \psi _{ij} - \phi _i) - \Omega _d^I \sin (\phi _i) + \Lambda _i(t), \end{aligned}$$

8 $$\begin{aligned} {\dot{\phi }}_i^A&= - \frac{\Omega _v^A}{N_{c,i}} \sum _{j \in VC} e^{-r_{ij}/R_0} \sin {(\psi _{ij} - \phi _i)} + \Lambda _i(t). \end{aligned}$$

### Parameters

In the simulation, we measure the length in units of $R_0$, and time in units of $\tau = 1/D_R$, where $D_R$ is the rotational diffusion coefficient. In order to guarantee an overdamped dynamics, where inertial effects are negligible, simulations are performed by selecting a sufficiently large friction coefficient $\gamma$, such that $mD_R/\gamma \ll 1$. Explicitly, we set $\gamma = 100 m D_R$, with $m=1$ and $D_R=1$. The inclusion of the inertia term serves to enhance the accuracy of the numerical integration process of Eq. 1. The size of the particles is chosen to be $\sigma = R_0/2$. The length *L* of the two-dimensional simulation box with periodic boundary conditions is taken to be $L/R_0=50$, large enough to avoid significant finite-size effects. The box size is used to control the packing fraction $\Phi = N \pi R_0^2/16L^2$. The system contains $N_I = 1$ intruder and $N_A = 6365$ agents, so that the total particle number is $N = N_I+N_A=6366$. The equations of motion are integrated by the velocity–Verlet scheme, employing a time step $\Delta t=2 \times 10^{-4}\tau$.

The activity of the iABPs is characterized by the dimensionless Péclet number *Pe*, which is the ratio of directed motion to diffusion, $Pe = v_0/(D_R R_0)$. In terms of the Péclet number, the active force becomes $f_a = \gamma v_0 = Pe \cdot \gamma D_R R_0$. The strength $\epsilon$ of the repulsive WCA potential is chosen to increase with increasing Péclet number, $\epsilon =(1+Pe)mR_0^2D_R^2$, such that the effective hard-core radius of the particles is not affected by activity.

The vision cones of intruder and agents are chosen to be distinct, determined by specifying their respective activities, vision angles, and vision ranges as shown in Table 1, in order to capture different properties of the two types of iABPs.

The effects of self-propulsion and self-steering are to some extent opposing each other. Higher propulsion speed implies smaller trajectory curvature (at fixed maneuverability), while higher maneuverability implies larger trajectory curvature (sharper turns). This competition can be captured by the parameter $\Delta = (Pe^{3/2})^A/\Omega _{v}^A$; it was shown in Ref.^35^ that for fixed $\Delta$ minimum distance distributions for moving self-avoiding agents in a crowd for various *Pe* and $\Omega _{v}$ collapse onto a single scaling curve. We choose $\Delta =0.5$, with vision-induced maneuverability $\Omega _v^A = 16$ and activity $Pe^A = 4$, corresponding to the “squirming” regime in Ref.^35^, where agents are able to steer away from their neighbors to avoid collisions while in motion. The packing fraction of agents is set to $\Phi =0.5$, in order to have a strong influence of the interaction between the intruder and the agents. All other parameters are maintained the same as listed in Table 1.

For pedestrians at walking speed, inertia effects are typically small, and we have therefore neglected them here. However, we expect on the basis of previous studies^38^ that inertia would reduce clustering. Inertia would also affect the ability for the redirection of motion, as it favors motion persistence; therefore, it reduces the ability of particles to redirect its motion, and thereby effectively reduces maneuverability^39^.

**Table 1:** Parameters of intruder and agents.

Parameter	Value	Definition
$Pe^{I}$	4	Péclet number of intruder
$Pe^{A}$	4	Péclet number of agents
$\theta ^{I}$	$\pi /4$	Vision angle of intruder
$\theta ^{A}$	$\pi /2$	Vision angle of agents
$R_{v}^{I,A}$	$2R_0$	Vision range intruder-to-agent
$R_{v}^{A,I}$	$4R_0$	Vision range agent-to-intruder
$R_{v}^{A,A}$	$4R_0$	Vision range agent-to-agent
$\Omega _d^{I}/D_R$	$10^{0}\text {-}10^{2}$	Directional maneuverability of intruder
$\Omega _v^{I}/D_R$	$10^{-1}\text {-}10^{3}$	Vision-induced maneuverability of intruder
$\Omega _v^{A}/D_R$	$10^{-1}\text {-}10^{3}$	Vision-induced maneuverability of agent
$\Omega _v^{A,I}/D_R$	$10^{-1}\text {-}10^{3}$	Distinct agent-to-intruder maneuverability

## Results

We investigate how the intruder’s ability to perform directed motion in a dense crowd of agents depends on the maneuverabilities of the intruder and agents, the directional maneuverability of the intruder to move in the goal direction, and the density (area fraction) of the agents. Three different scenarios for the interactions between intruder and agents are considered: (i) The intruder avoids nearby agents through vision-induced maneuverability $\Omega _v^I$, thereby preventing collisions, with the goal to enhance the directed motion. (ii) The agents employ their own vision-induced maneuverability $\Omega _v^A$ to avoid collisions with both the intruder and other agents. (iii) The agents exhibit a distinct, stronger vision-based maneuverability $\Omega _v^{A, I}$ to avoid collisions with the intruder, compared to other agents, enabling them to avoid the vicinity of the intruder more efficiently.

**Fig. 2:**
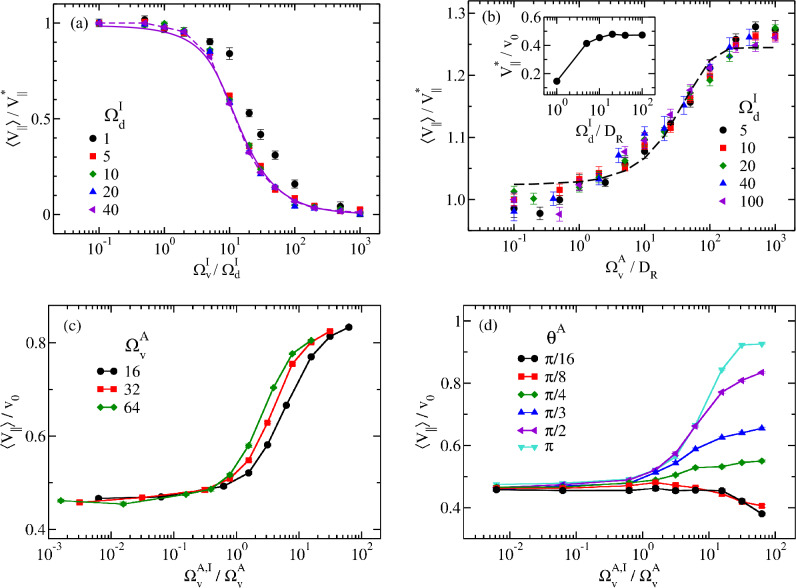
Average directed speed $\langle V_\parallel \rangle$ of an intruder. (**a**) As a function of the intruder’s maneuverability ratio $\Omega _v^I/\Omega _d^I$, with the agent’s maneuverability $\Omega _v^A=16$ and for various directed maneuverabilities $\Omega _d^I = 1$, 5, 10, 20, 40 of the intruder. The reference speed $V_\parallel ^*$ is the intruder’s speed for vanishing vision-induced maneuverability ($\Omega _v^I/\Omega _d^I = 10^{-1}$) for $\Phi =0.5$. The simulation results (dashed line) show good agreement with the theoretical predictions (solid lines) obtained from Eq. (11). (**b**) As a function of the agent’s vision maneuverability $\Omega _v^A$ for various $\Omega _d^I = 5,10,20,$ and 40, at $Pe^I = Pe^A = 4$. Inset: The reference speed $V_\parallel ^*$ increases with increasing directional maneuverability of the intruder and saturates for $\Omega _d^I \gtrsim 5$. It is normalized by the unperturbed intruder speed, $v_0=Pe^I = 4$. (**c**) As a function of agents-to-intruder maneuverability $\Omega _v^{A,I}$ for the intruder’s vision and directed maneuverability $\Omega _v^I =16$ and $\Omega _d^I=10$, respectively, with agent-to-agent vision maneuverability $\Omega _v^A=16$ and agent activity $Pe^A=4$. The reference speed $v_0$ is the unperturbed intruder speed, $v_0=Pe^I = 4$. (**d**) As a function of agent-to-intruder maneuverability $\Omega _v^{A,I}$, for various vision angles of agents in the range $\theta ^A\in (\pi /16,\pi )$ for $\Omega _v^A=16$. The intruder’s vision and directed maneuverability are $\Omega _v^I=16$ and $\Omega _d^I=10$, respectively. The reference speed $v_0$ is the unperturbed intruder speed, $v_0=Pe^I = 4$.

**Fig. 3:**
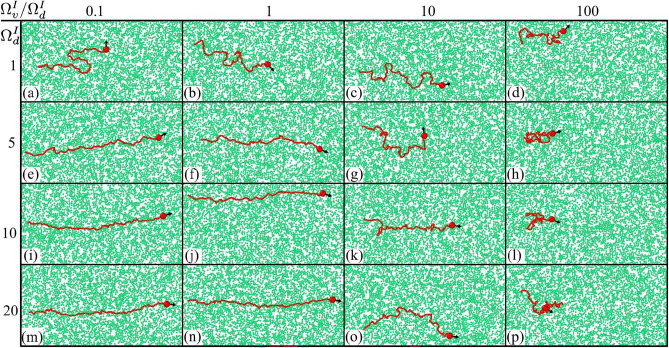
Trajectories of the intruder (red) moving through a dense crowd of agents (green), at area faction $\Phi =0.5$. For clarity, the intruder is illustrated at a larger size; its actual size is identical to that of the agents. The respective simulation videos are shown in the supplementary movies.

### Directed speed of the intruder

#### Effect of intruder’s maneuverability

We investigate the role of the intruder’s maneuverability on the dynamics of the intruder while fixing various parameters that control the agents’ dynamical behavior.

Figure 2a shows the normalized average directed speed of the intruder, $\langle V_\parallel \rangle / V_\parallel ^*$, as a function of the intruder’s maneuverability ratio, $\Omega _v^I / \Omega _d^I$. For small $\Omega _v^I / \Omega _d^I \lesssim 3$, goal-directed maneuverability dominates over the vision-induced maneuverability. In this regime, the intruder hardly reacts to the presence of the agents by steering, and is therefore not distracted by their presence from its motion toward the goal direction - except for collisions due to volume exclusion. Once $\Omega _v^I / \Omega _d^I \gtrsim 5$, vision-induced avoidance becomes significant, leading to a gradual decrease in speed; the drop becomes more pronounced for $\Omega _v^I / \Omega _d^I \gtrsim 10$. This decrease indicates the intruder’s limited capability to maintain its intended direction as it attempts to avoid collisions with the agents by steering. For very large $\Omega _v^I / \Omega _d^I > 100$, the avoidance steering overwhelms goal alignment, causing the directional speed to approach zero. The speed of the intruder is controlled by two mechanisms: (i) the competition of directional motion and rotational diffusion in the absence of vision-induced reorientation, and (ii) the competition between directional motion and vision-induced avoidance. The first mechanism can be calculated analytically by analysis of the Fokker-Planck equation (see Supplementary Information (SI), Sec. S1 A), which yields^40^

9 $$\begin{aligned} \langle V_\parallel \rangle = v_0 \, \langle \cos (\phi ) \rangle = v_0 \, I_1(\Omega _d^I/D_R)/I_0(\Omega _d^I/D_R) \, \end{aligned}$$

where $I_n(...)$ is the modified Bessel function of the first kind.

The second mechanism is more difficult to predict, as it arises from a multi-body interaction. However, it is clear that the avoidance steering implies frequent and significant changes of the direction of motion and thus of the particle orientation. On a mean-field level, this effect can be captured by an effective diffusion coefficient $D_{eff}^I$, which increases proportional to the vision-induced maneuverability, i.e.

10 $$\begin{aligned} D_\text {eff}^I = D_R + g(\Phi ){\Omega _v^I} \end{aligned}$$

with an agent-density-dependent proportionality factor $g(\Phi )$ with $g(0)=0$.

With this assumption about the effect the visual maneuverability, we obtain a scaling expression analogous to Eq. (9) (see SI, Sec. S2 A) of the form

11 $$\begin{aligned} \langle V_\parallel \rangle = v_0 (\Phi ) \, \frac{I_1(\Omega _d^I/D_\text {eff}^I)}{I_0(\Omega _d^I/D_\text {eff}^I)}, \end{aligned}$$

Here, $v_0(\Phi )$ is the density-dependent intruder speed at $\Omega _v^I=0$. A comparison of this relation with the simulation data in Figs. 2a and 5a gives an excellent agreement for a choice ${g(\Phi )}\simeq 0.06$ for area fractions $\Phi$ in the range $0.2 \lesssim \Phi \le 0.5$. Thus, we conclude that the vision-related maneuverability of the intruder indeed acts as an effective noise. For small $\Phi$, however this scaling breaks down (see Section Role of agent density below). This is consistent with expectations, as the mean-field, effective-noise description of vision-induced steering is only valid when the intruder perceives several surrounding agents. At low densities, avoidance motion becomes strongly correlated, as individual steering maneuvers are separated by longer periods without interactions, which invalidates the random-noise approximation.

Our results reveal that vision-induced maneuverability decreases the intruder’s efficiency to perform directed movement. This is intuitively surprising, as the ability to avoid collisions with obstacles (agents) would seem to prevent collision-induced slowdown and thus increase the directional speed. However, the detours due to collision avoidance obviously outweigh these gains. A look at the intruder trajectories for various directional and visual maneuverabilities, see Fig. 3, nicely demonstrates the increasing tortuosity of the intruder’s path with increasing $\Omega _v^I$ as it tries to curve around agents, and thereby looses more and more its determination to reach the goal.

Interestingly, the directional speed of the intruder is nearly independent of the sign of the visual maneuverability, i.e. whether the intruder steers away or toward regions of higher agent density, see SI, Sec. S2 B and Fig. S5. This symmetry arises because steering toward regions of higher or lower agent density has essentially the same effect of distracting the intruder from its goal-oriented motion.

#### Effect of agent maneuverability

An interesting aspect of a crowd of intelligent active particles is that the agent’s maneuverability also affects the dynamics of the intruder. In order to examine this effect, we fix the vision maneuverability of the intruder $\Omega _v^I = 16$ while keeping all other parameters for both the agents and the intruder the same as listed in Table 1 at $\Phi = 0.5$.

Figure 2b shows the variation of the intruder’s normalized average directed speed, $\langle V_\parallel \rangle / V_\parallel ^*$, as a function of the agent’s vision-induced maneuverability, $\Omega _v^A$. Interestingly, increasing $\Omega _v^A$ leads to a pronounced enhancement of the intruder’s average directed speed. $\langle V_\parallel \rangle$ increases monotonically with $\Omega _v^A$ and saturates for large maneuverability, $\Omega _v^A \gtrsim 10^3$, corresponding to a nearly $30\%$ enhancement. Remarkably, when $\langle V_\parallel \rangle$ is scaled by the intruder speed $V_\parallel ^*$ for small $\Omega _v^A \lesssim 1$, the relative directed speeds for all investigated goal fixations $\Omega _d^I$ collapse onto a single universal curve. This implies that the vision-based maneuverability of the agent strongly influences the ability of the intruder to maintain persistent motion. Taken together with the results from Section Effect of Intruder’s maneuverability, we conclude that an intruder achieves the largest directed motion when it does not attempt to avoid collisions; instead, it is the bath of agents that must avoid the oncoming intruder. For low agent maneuverability $\Omega _v^A \lesssim 1$, excluded-volume effects dominate the dynamics, leading to frequent intruder-agent and agent-agent collisions that restrict the intruder’s directed speed and cause it to saturate.

As $\Omega _v^A$ increases, vision-induced avoidance becomes prominent, enabling agents to steer clear of both their neighbors and the intruder, thereby reducing collisions with the intruder and promoting more efficient intruder motion. At large $\Omega _v^A \gtrsim 10^3$, the finite area fraction constrains the agent’s ability to maintain large mutual separation, preventing further suppression of collisions. Consequently, the intruder’s directed speed saturates in this regime (see Fig. 2b).

The inset of Fig. 2b shows that for small $\Omega _v^A$ the speed $V_\parallel ^*$ of the intruder saturates for $\Omega _d^I \gtrsim 10$, which implies that a further increase of $\Omega _d^I$ does not generate an additional increase of the intruder’s average directed speed. In this regime, the intruder’s propulsion efficiency is limited by excluded-volume collisions with the agents ($\Phi =0.5$), so that $V_\parallel ^*/v_0 \simeq 0.5$. This diminishing sensitivity to $\Omega _d^I$ is also consistent with the result shown in SI, Sec. S1 C, Fig. S3, where the alignment of an isolated intruder is found to saturate beyond $\Omega _d^I\gtrsim 10$.

**Fig. 4:**
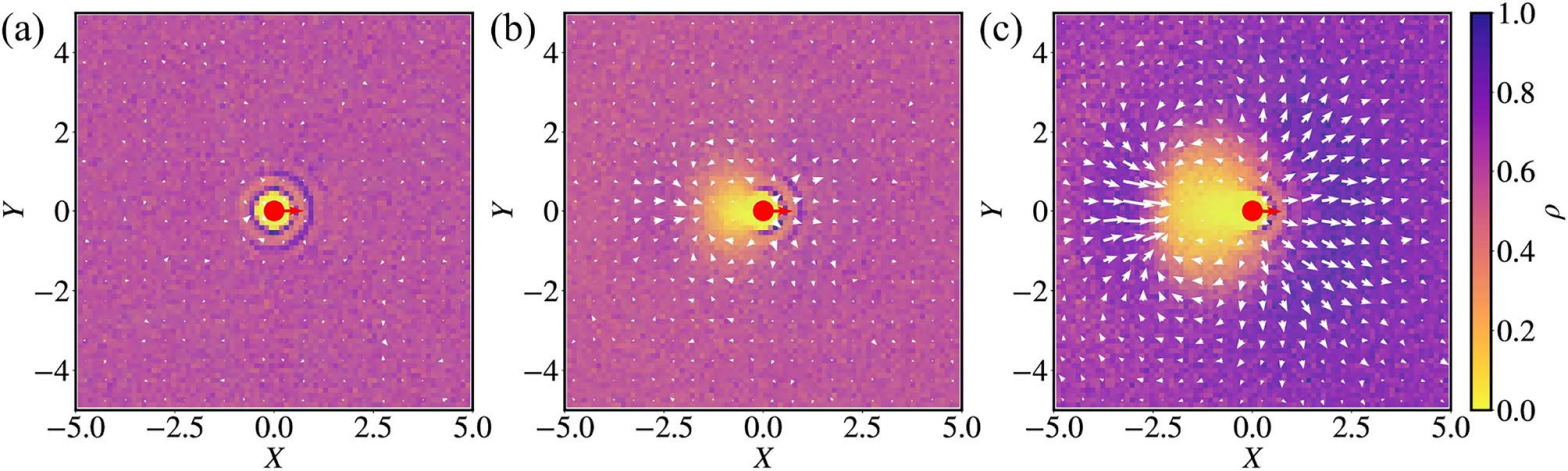
Local agent density around the intruder for increasing maneuverability. Density colormaps illustrate the effect of agent maneuverability at $\Omega _v^{A,I} / \Omega _v^A = 0.625$, 6.25, and 31.25 (left to right) for the intruder’s directed maneuverability $\Omega _d^I = 10$. The colorbar denotes the normalized agent density. The red arrow marks the intruder’s orientation, while white arrows indicate the surrounding agents’ velocity field.

#### Distinct Agent-Intruder Maneuverability

Since the intruder differs in its identity from the agents, it is also interesting to consider different reactions of the agents to the presence of the intruder than to other agents. To do this, we distinguish the agent-to-intruder maneuverability, $\Omega _v^{A,I}$, from the inter-agent maneuverability, $\Omega _v^A$.

Figure 2c displays the simulation results for the average directed speed of the intruder as a function of the agents-to-intruder maneuverability $\Omega _v^{A,I}$, for various $\Omega _v^A$ values. Interestingly, increasing $\Omega _v^{A,I}$ leads to a pronounced increase of the intruder’s directional speed, $\langle V_\parallel \rangle / V_0$, significantly larger than that observed for increasing $\Omega _v^A$, compare Fig. 2b. Also, the curves for various $\Omega _v^A$ nearly coincide, which indicates that the agent-agent avoidance steering is of minor importance in this case. This conclusion is supported by the fact that the pronounced increase of $\langle V_\parallel \rangle$ in Fig. 2c occurs when $\Omega _v^{A,I} \gtrsim \Omega _v^A$. Furthermore, the increase in inter-agent maneuverability $\Omega _v^A$ results in a slight reduction in the intruder’s directed relative speed - because stronger agent-agent avoidance pushes neighboring agents toward the intruder. This increases the crowding of agents around the intruder, which hinders its motion.

To further elucidate the underlying mechanism, we calculate the density distribution of the agents around the intruder, in the intruder’s body-fixed reference frame. The results in Fig. 4 for three visual maneuverabilities $\Omega _v^{A,I}/\Omega _v^A$ = 0.625, 6.25, and 31.25 display a clear depletion region behind the intruder, which grows in size and becomes more pronounced with increasing $\Omega _v^{A,I}/\Omega _v^A$ due to the enhanced avoidance steering of the agents. In addition, the agent density in front of the intruder increases, because agents in a dense crowd cannot move away side-wise quickly enough. Figure 4 also displays the local velocity field of the agents, which demonstrates that agents move avoid the intruder in part by escaping ahead of it in the goal direction, but also to a large part by escaping side-wise. A reverse side-wise motion behind the intruder again closes the void, so that the void is actually traveling together with the intruder. The reduction in local agent density effectively facilitates the intruder’s movement with reduced interference from surrounding agents. For a more detailed discussion of this ‘void’ region, in particular the dependence of its size on $\Omega _v^{A,I}/\Omega _v^A$, see Section Agent-depleted ‘void’ region around intruder below.

#### Role of agent density

All results presented so far are obtained for a fixed agent packing fraction $\Phi =0.5$. To elucidate the role of agent density, we next examine the dependence of the intruder’s dynamics on $\Phi$. Increasing $\Phi$ increases the frequency of intruder–agent interactions, hence it should result in a slow-down of the intruder dynamics.

**Fig. 5:**
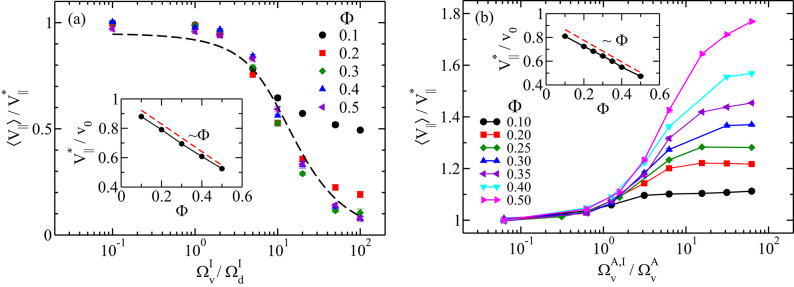
Dependence of the normalized directional speed of the intruder on agent density. (**a**) As a function of the $\Omega _v^I/\Omega _d^I$ for various packing fractions $\Phi \in (0.1,0.5)$ at $\Omega _v^A = 16$ and $\Omega _d^I = 10$. The normalization factor is the upper plateau value $V_\parallel ^*$ for $\Omega _v^{A,I} = 10^{-1}$. The dashed line shows theoretical fit at $\Phi = 0.5$. The inset shows the linear behavior of $V_\parallel ^*$ with $\Phi$. (**b**) As a function $\Omega _v^{A,I}/\Omega _v^A$ for $\Phi \in (0.1,0.5)$, at $\Omega _v^A = 16$, $\Omega _d^I = 10$, and $\Omega _v^I = 16$. Reference speed $V_\parallel ^*$ is the lower plateau value for $\Omega _v^{A,I} = 1$. The inset illustrates that the plateau value of the intruder’s directed speed $V_\parallel ^*$ linearly decreases as the agent density $\Phi$ increases.

The results for the intruder’s directional speed as a function of $\Omega _v^I/\Omega _d^I$, displayed in Fig. 5a, when scaled by the speed $V_\parallel ^*$ at vanishing visual maneuverability of the intruder, show a good data collapse for semi-dense and dense systems with agent area fractions $\Phi \gtrsim 0.2$. The speed decreases from its plateau value at $\Omega _v^I \rightarrow 0$ with increasing $\Omega _v^I/\Omega _d^I$ and asymptotically approaches another plateau at large $\Omega _v^I$, similar to the behavior in Fig. 2a. Deviations from this universal behavior are found in the dilute case $\Phi = 0.1$, where intruder–agent collisions are weak, and the reduction in directional speed is comparatively weaker with increasing $\Omega _v^I/\Omega _d^I$. The inset of Fig. 5a shows low-$\Omega _v^I$ speed $V_\parallel ^*$ decreases linearly with $\Phi$,

12 $$\begin{aligned} v(\Phi | \Omega _v^I=0) = v_0 \left( 1 - \frac{\Phi }{\Phi _0}\right) , \end{aligned}$$

with $v_0 \approx 3.87$, consistent with the speed $v_0$ of an isolated intruder, and $\Phi _0 \approx 1.08$ obtained from linear fitting.

Figure 5b presents the dependence of the intruder’s normalized directed speed on the agent-to-intruder maneuverability ratio for several packing fractions, $\Phi \in (0.1, 0.5)$. For small $\Omega _v^{A,I}/\Omega _v^A$, the density dependence of the speed is again well captured by Eq. (12). A significant additional dependence on $\Phi$ is found for $\Omega _v^{A,I}/\Omega _v^A \gtrsim 1$, in general agreement with the results of Section Effect of agent maneuverability. Interestingly, but maybe also not very surprisingly, the intruder benefits from the avoidance steering of the agents most at high agent densities. This benefit decreases with decreasing density. For dilute suspensions, the abundance of free space makes the intruder’s speed nearly independent of its steering capabilities.

#### Influence of vision angle of agents

The intruder’s vision range is considered here to be smaller than that of the agents - motivated by the desired directional motion and thus more focused vision in the forward direction. The agents, on the other hand, can respond to the intruder from a larger distance. Therefore, only the variation of the vision angle $\theta ^A$ of the agents is considered, and its influence on the motion of the intruder is examined.

Figure 2d shows that the intruder’s directional speed remains nearly unchanged across all $\theta ^A$ for $\Omega _v^{A,I} \lesssim 10$. However, for larger $\Omega _v^{A,I}$, the average directed speed $\langle V_\parallel \rangle$ is found to strongly depend on $\theta ^A$. For large vision angles $\theta ^A \gtrsim \pi /4$, the intruder speed displays a pronounced increase, whereas for narrow vision angles $\theta ^A \le \pi /8$, the directed speed is reduced. A wide field of view allows the agents to detect the intruder earlier, facilitating avoidance and the formation of a void region around it, which helps the intruder maintain a higher directed speed. Conversely, a narrow field of view limits detection efficiency, increases collision frequency, and ultimately reduces the intruder’s directed speed.

**Fig. 6:**
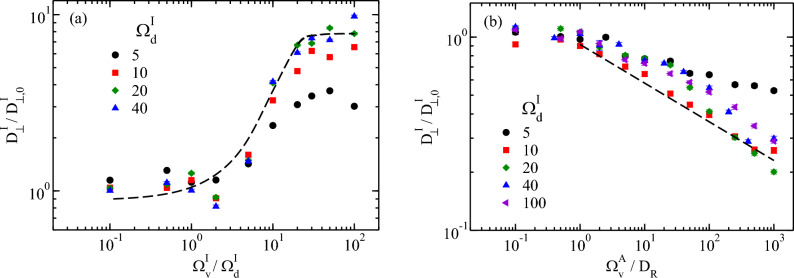
Normalized transverse diffusivity $D_\perp ^I/D_{\perp ,0}^I$ of the intruder. (a) As a function of the intruder’s maneuverability ratio $\Omega _v^I/\Omega _d^I$ for three directional maneuverabilities $\Omega _d^I = 5$, 10, 20, and 40, with agent maneuverability $\Omega _v^A = 16$. (b) As a function of $\Omega _v^A$ for four distinct values of the intruder’s directional maneuverability, $\Omega _d^I = 5$, 10, 20 and 40, with $\Omega _v^I = 16$. In both (a) and (b), the transverse diffusivity is scaled by the diffusivity coefficient $D_{\perp ,0}^I$ at $\Omega _v^A=0$; dashed lines are guides to the eye.

### Intruder’s transverse diffusivity

The transverse diffusivity $D_\perp ^I$ of the intruder provides information about the deviations of its trajectory from a straight path in the goal direction. We calculate the mean-square displacement (MSD) in the transverse direction,

13 $$\begin{aligned} \langle \Delta r_\perp ^2(t) \rangle = \langle |r_\perp (t+\tau ) - r_\perp (\tau )|^2 \rangle \end{aligned}$$

as a function of the intruder’s maneuverability ratio. Here, the average is calculated over the initial time $\tau$.

The intruder performs directed motion, and thereby MSD in its goal (*x*) direction exhibits a ballistic nature for long times. Conversely, in the transverse direction, the MSD attains diffusive behavior. The transverse diffusion coefficient $D_\perp ^I$ in the diffusive regime is obtained from the MSD as^41^,

14 $$\begin{aligned} D_\perp ^I = \lim _{t \rightarrow \infty } \frac{1}{2} \frac{d}{dt} \langle \Delta r_\perp ^2(t)\rangle . \end{aligned}$$

As a reference case, it is again interesting to consider the motion of an intruder with directional maneuverability, but with vanishing visual maneuverability. In this case, the analysis of the Fokker-Planck equation (see SI, Sec. S1 B), for $\Omega _d^I \gg D_R$ yields the analytical result for the transverse diffusivity

15 $$\begin{aligned} D_{\perp ,0}^I = \frac{v_0^2 D_R}{4(\Omega _d^I)^2}. \end{aligned}$$

This result can be compared with the activity-induced translational diffusivity of a simple, non-steering ABP, which is given by $D_t^{(ABP)}=v_0^2/(2D_R)$. The comparison shows that $D_{\perp ,0}^I = D_t^{(ABP)} (D_R/(2\Omega _d^I))^2$. Thus, the goal fixation strongly reduces the diffusivity perpendicular to the goal direction (for $\Omega _d^I \gg D_R$).

Figure 6a shows the normalized transverse diffusivity $D_\perp ^I/D_{\perp ,0}^I$ of the intruder as a function of the maneuverability ratio $\Omega _v^I / \Omega _d^I$, relative to the reference diffusivity $D_{\perp ,0}^I$ without visual maneuverability. For small $\Omega _v^I / \Omega _d^I$, $D_\perp ^I/D_{\perp ,0}^I$ remains nearly independent of the visual maneuverability, consistent with the constant-speed regime in Fig. 2a. For $\Omega _v^I / \Omega _d^I \gtrsim 5$, $D_\perp ^I/D_{\perp ,0}^I$ increases monotonically, while the directional speed decreases, compare Fig. 2a. The transverse diffusivity levels off at $\Omega _v^I / \Omega _d^I \gtrsim 25$. At larger $\Omega _v^I / \Omega _d^I$, vision-induced avoidance dominates because directional maneuverability saturates for $\Omega _d^I \gtrsim 5$ (see SI, Fig. S3), whereas vision-based maneuverability continues to induce deflections from the goal-directed motion. As a result, the intruder loses directional persistence, and its trajectory becomes increasingly random.

Interestingly, the transverse diffusivity $D_\perp ^I/D_{\perp ,0}^I$ displays a very similar dependence on $\Omega _v^I / \Omega _d^I$ for all $\Omega _d^I \gtrsim 10$. Furthermore, it displays a similar, but inverse, dependence on $\Omega _v^I / \Omega _d^I$ as the directional speed $\langle V_\parallel \rangle /v_0$. This suggests that the same physical mechanism underlies these behaviors. This hypothesis can be substantiated by a calculation with a simplified toy model, see SI Sec. S2 A), which suggests

16 $$\begin{aligned} D_\perp ^I = D_T^{(ABP)} \left(1-\frac{\langle V_\parallel \rangle}{v_0}\right)^2 \end{aligned}$$

compare SI, Eq. (S18). Equation (16) makes plausible that in the presence of vision-induced avoidance steering implies the same, but inverse, functional dependence of $D_\perp ^I$ on $\Omega _v^I/\Omega _d^I$ as the directional speed $\langle V_\parallel \rangle$, which applies, in particular to location of the crossover the transition from goal-direction dominated to visual-avoidance dominated motion at $\Omega _v^I/\Omega _d^I\simeq 10$.

We also examine the transverse diffusion $D_\perp ^I/D_{\perp ,0}^I$ of the intruder as a function of the agent’s vision maneuverability $\Omega _v^A$, see Fig. 6b. In contrast to its increase with the intruder’s maneuverability, the diffusivity slowly decreases with increasing $\Omega _v^A$. For $\Omega _d^I \gtrsim 10$, the relative change in $D_\perp ^I/D_{\perp ,0}^I$ becomes nearly independent of $\Omega _d^I$. The reduction of transverse diffusion with increasing agent visual maneuverability aligns with the enhancement in the intruder’s average directed speed, see Fig. 2b, as in both cases the reduced agent density near the intruder due to agent steering implies less collisions and less avoidance steering by the intruder.

In summary, transverse diffusion and directional speed of the intruder are closely (and inversely) correlated, because both are related to deviations from straight motion in the goal direction - which reduce directional speed and enhance transverse diffusivity. These deviations are caused by the vision-induced avoidance steering of the intruder. Conversely, increased directed maneuverability $\Omega _d^I$ of the intruder, as well as increased vision-controlled avoidance by the agents, which depletes the agents in the vicinity of the intruder, promote goal alignment and suppress transverse diffusion.

**Fig. 7:**
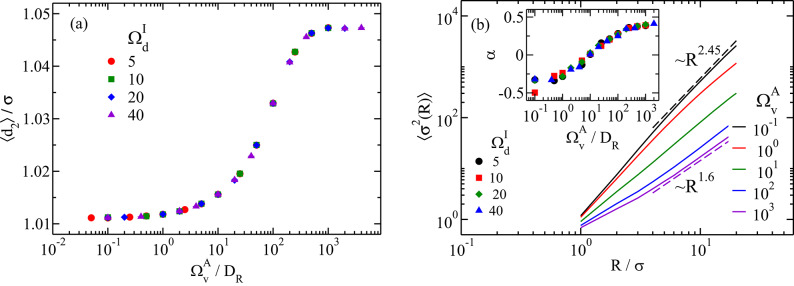
(**a**) Average minimal neighbor distance $\langle d_2 \rangle$ between agents as a function of agent visual avoidance maneuverability $\Omega _v^A$, at various directional maneuverabilities $\Omega _{d}^I=5$, 10, 20 and 40 of the intruder, for packing fraction $\Phi =0.5$. (**b**) Number variance $\langle \sigma _N^2(R) \rangle$ of the agents’ density distribution for various $\Omega _v^A$, with $\Omega _d^I=5,10,20,40$. The inset shows the resulting hyperuniformity exponent $\alpha$, which indicates a transition from a clustered state ($\alpha <0$) to a weakly hyperuniform state ($\alpha >0$) with increasing agent’s visual maneuverability $\Omega _v^A$, for $\Omega _d^I = 10$. .

### The agent crowd: minimal agent-agent distance, hyperuniformity, and void size around intruder

#### Average minimal agent-agent distance

We compute the average minimal neighbor distance $\langle d_2 \rangle / \sigma$ between agents within their vision cones, averaged over time.

Figure 7a shows the variation of $\langle d_2 \rangle / \sigma$ with $\Omega _v^A$. The minimal distance exhibits a sigmoidal-like increase and eventually saturates at large visual maneuverabilities, $\Omega _v^A > 10^2$. Moreover, $\langle d_2 \rangle$ is independent of $\Omega _d^I$, which is not too surprising as $\langle d_2 \rangle$ is a property of the agent crowd, and should thus be unaffected by the intruder. With increasing vision-induced self-steering, the agents can avoid other agents more effectively, which implies an increase in the average inter-agent distance. The increase of the average minimal distance is limited by the area fraction $\Phi$ of the agents, which corresponds to an average distance $d_\Phi = a_\Phi \Phi ^{-1/2}$. Here, $a_{\Phi }\approx 0.67$ is obtained by fitting the simulation data, see SI, Sec. S3, Fig. S6. This value agrees very well with the analytical result for a regular triangular lattice, $a_\Phi ^\Delta =\sigma \pi ^{1/2} 3^{-1/4}=0.67$, see SI, Sec. S3, Eq. (S22). This implies that the agent distribution is rather uniform, as characterized in more detail in the next section.

#### Hyperuniformity

The increase of the average minimal distance due to self-steering implies that the agents are not randomly (Poisson) distributed, but have a more homogeneous spatial distribution.

The degree of uniformity of the particle distribution can be characterized by examining density fluctuations over a range of length scales larger than the particle size. A system is denoted as “hyperuniform” if the variance in the number of particles within a spherical observation window of radius *R* grows more slowly than the observation volume with increasing *R*^42, 43^. Hyperuniformity provides a unifying framework for classifying and comparing a broad spectrum of structures, encompassing crystals, quasi-crystals, and disordered materials; thereby, it bridges the gap between ordered and disordered systems by a common statistical description^43, 44^.

The number variance $\sigma _N^2(R)$ of the agents in a circle of radius *R* is

17 $$\begin{aligned} \sigma _N^2(R) = \langle N(R)^2 \rangle - \langle N(R) \rangle ^2, \end{aligned}$$

where *N*(*R*) is the number of particles, and $\langle \cdot \rangle$ denotes the ensemble average. The number variance exhibits the asymptotic scaling behavior^43^ $\sigma^2_N(R) \sim R^{d-\alpha }$. Here, *d* is the spatial dimensionality and $\alpha$ a positive exponent, which characterizes the suppression of density fluctuations at larger length scales. Here, $\alpha >1$, $\alpha =1$, and $\alpha <1$ correspond to the fastest (Class I), intermediate (Class II), and slowest (Class III) decay of density fluctuations, respectively.

Figure 7b illustrates that in the crowd of agents with self-steering avoidance, the “hyperuniformity” exponent $\alpha$ increases with increasing maneuverability $\Omega _v^A$, indicating a transition from a clustered state ($\alpha <0$) for $\Omega _v^A \lesssim 10$ to a hyperuniform state for $\Omega _v^A \gtrsim 10$. The largest value of $\alpha \simeq 0.4$ signals a state with weak hyperuniformity (Class III). Note that the ‘critical’ value $\Omega _v^A \simeq 10$ roughly coincides with the beginning of the increase of average minimum distance $\langle d_2 \rangle$ in Fig. 7a. A similar behavior is obtained for the dependence of uniformity of the crowd of agents on the vision angle $\theta ^A$, where the “hyperuniformity” exponent $\alpha$ increases with increasing vision angle, and reaches $\alpha \simeq 0.1$ for $\theta ^A=\pi$ (with $\Omega _v^A=16$), as agents respond to the presence of all nearby neighbors (see SI, Sec. S5 and Fig. S8 for details).

More importantly, the ‘critical’ agent maneuverability also relates to changes in the behavior of an intruder. It matches with the agent maneuverability at which the intruder directional speed displays the most pronounced increase in Fig. 2b, and roughly agrees with the agent maneuverability where the change of the transverse diffusivity of the intruder is seen from nearly independent of $\Omega _v^A$ to a reduced diffusivity in Fig. 6b. Thus, a more uniform agent distribution facilitates an easier navigation of the intruder through the active environment, resulting in an increased average directional speed and reduced transverse diffusivity.

It is important to note that the hyperuniformity exponent $\alpha$ is extracted in Fig. 7b from data for window sizes $5 \le R/\sigma \le 25$, which seems appropriate for the comparison with the intruder directional speed. However, these window sizes are too small to allow conclusions for the asymptotic behavior. Therefore, we also present simulation results for larger window sizes, which confirm the results of Fig. 2b for large $\Omega _v^A/D_R$, showing that the system becomes asymptotically weakly hyperuniform. Additionally, for small $\Omega _v^A/D_R$, where the agents are hardly interacting, asymptotically $\alpha \simeq 0$, i.e. a Poisson-like random distribution. See SI, Sec. S5 and Fig. S9 for details.

**Fig. 8:**
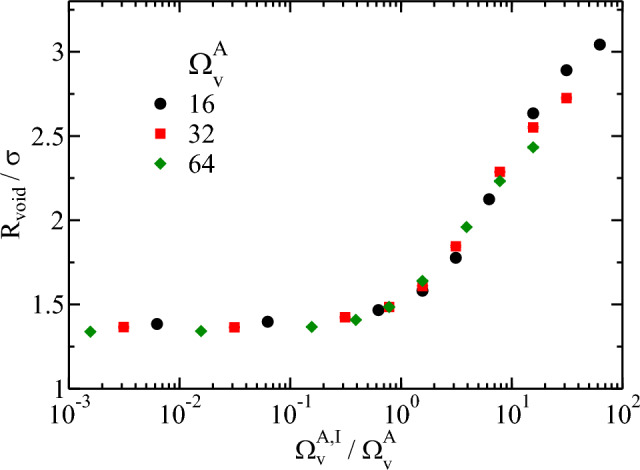
The variation of the average void radius $R_{void}$ around the intruder as a function of the agent-to-intruder maneuverability, with $\Omega _v^A=16$, 32 and 64.

#### Agent-depleted ‘void’ region around intruder

The stronger the avoidance of the intruder by the agents, the larger the region of reduced agent density in the vicinity of the intruder, as indicated by the agent distribution around the intruder in the intruder’s reference frame, displayed in Fig. 4, and by the pronounced increase of the intruder directional speed with increasing agent-to-intruder maneuverability in Section Role of agent density, see Fig. 5b.

To quantify the size of this void, we identify the nearest-neighbor agents in front and behind the intruder. The nearest agents within these domains define the front and rear neighboring distances, which together provide an estimate of the void radius $R_{void}$. See SI, Sec. S4 and Fig. S7, for details.

Figure 8 quantifies the growth of the size of the void region around the intruder with increasing agent-to-intruder maneuverability $\Omega _{v}^{A,I}$, which is already illustrated by the agent density maps in Fig. 4. This increased void size is indicative of two main effects, which contribute to the increased directional speed of the intruder (compare Fig. 4): (i) a reduced agent density in front of the intruder, and (ii) a longer-range flow of the agents co-moving with the intruder, with the highest speed in front of the intruder. Both effects imply a significant reduction of direct collisions due to volume exclusion.

Another important observation is that an increase in inter-agent vision maneuverability, $\Omega _v^A$, leads to a decrease in the void radius, as anticipated in Sec. 3.1. This behavior arises from inter-agent avoidance, which promotes uniformity within the system and thus results in an inflow of agents *back* into the void as they steer away from other agents outside the void. The comparison of the average directional speed of the intruder in Fig. 2c and the void radius in Fig. 8 shows that there is indeed a strong correlation between $\langle V_\parallel \rangle$ and $R_{void}$.

## Summary, discussion, and conclusions

We have studied the directed motion of an intruder in a crowd of identical active agents by overdamped Langevin dynamics simulations. Both intruder and agents are modelled as intelligent active Brownian particles (iABPs) with visual perception and directional steering to avoid collisions. Intruder and agents can have different vision angles and maneuverabilities. Our results elucidate the dependence of the directional speed of the intruder on key control parameters, like maneuverability, vision angle, and agent density.

The maneuverability associated with goal orientation promotes efficient motion toward the target. However, while vision-induced intruder maneuverability helps to avoid close encounters or collisions with agents, the expected improvement in directional speed does not materialize. Instead, avoidance steering drives large deviations from the beeline and effectively increases particle diffusion, as indicated by the strong rise in transverse diffusivity. In contrast, a stronger avoidance response of the agents to the presence of the intruder creates a region of low agent density around the intruder, which facilitates an efficient directional motion of the intruder.

Thus, our main conclusion is that the fast directional motion of an intruder in a crowd depends much more on the “collaboration” of the crowd to clear a path through the crowd than on the intruder to curve around individual obstacles. Thus, it is more important for the intruder to make the agents in the crowd aware of its presence by enhancing its visibility than to actively avoid collisions by steering. This conclusion is in excellent agreement with real-world examples, such as an ambulance moving through a crowd of pedestrians, with the siren wailing (long-distance signaling) and the lights flashing (short-distance signaling)^13^. Thus, it would be interesting to perform experiments with human intruders in a pedestrian crowd, where the intruder employs various levels of optical and acoustic signals to make the crowd aware of its presence and directional motion. However, for an experimental test of our predictions, and a system of programmable microbots, such as a system of light-controlled, self-propelling colloidal particles^25, 45^ or of swarmbots^46^ would be better suited, simply because their properties and behavior can be much better controlled.

The description of perception-based self-steering motion in our model is fundamentally different from that of interactions in force-based models, in particular the social-force model^47^, in which interactions are implemented by repulsive forces that decay exponentially with distance. Experiments with an intruder moving through a crowd of pedestrians demonstrate that induced motion does not originate from mechanical interactions - like for an intruder moving through a granular medium -, but pedestrians anticipate the intruder’s passage by moving significantly before contact, and their displacements are thus mostly lateral, hence not aligned with the forces exerted by the intruder^11^. This leads to depletion zone behind *and* in front of the intruder. In our model, the agents anticipate the presence of the intruder and steer to avoid collisions - however, the implied lateral motion of the crowd (compare Fig. 4) is not yet strong enough to generate a depletion zone in front of the intruder. This implies that the agents in our model require additional cognitive capabilities to anticipate the *motion* of the intruder rather than its mere presence. This can be included in our model along the lines presented in Ref.^36^, where agents are able to distinguish the direction of motion (oncoming/comoving) of other particles. This could be an alternative to providing agents with the ability for planning a global strategy that minimizes their overall discomfort^48^.

Furthermore, we have shown that the intruder’s directional speed decreases linearly with increasing agent density, analogous to the collision-induced slowdown observed in standard ABP systems^49^. The directed speed also depends strongly on the vision angle of the agents, with a crossover from increasing to decreasing speed as agents transition from a larger to a smaller field of vision with increasing agents’ maneuverability. Notably, the intruder benefits most from the agents’ responsiveness in a crowd, where they respond more aggressively to the intruder than to other agents.

Although the reaction of a crowd to an intruder, where both particle types self-steer to avoid collisions, is phenomenologically similar to the reaction of a herd (or swarm) of prey to a predator^37, 50^, it is important to note that the two systems are actually quite different, because in the latter case the predator is chasing the prey, whereas in the former case the intruder aims to reach an “externally” determined goal.

Our results indicate that hyperuniformity is an important parameter that determines the penetrability of a crowd. Hyperuniformity is found to be strongly affected by the agents’ activity and avoidance steering. Agents avoid clustering and keep their distance from others due to self-steering, while avoiding crystallization due to orientational noise and diffusion. Such a state is more susceptible to opening up a path for the intruder due to its fluidity and active responsiveness.

It has been demonstrated that several other active systems display hyperuniform conformations - such as algae suspensions (with $\alpha =0.6$, where long-range hydrodynamic interactions dominate)^51^, and chiral pear-shaped Quincke rollers (with $\alpha =0.25 \dots 0.5$, where uniformity increases with curvature radius of their quasi-circular motion)^52, 53^. In these systems, particles are self-propelled or actuated, and are governed by magnetic, steric, or hydrodynamic interactions, but lack sensing and self-steering capabilities; i.e., they are “dumb” in our notation.

In contrast, we have presented here result for the hyperuniformity of systems of “intelligent” particles. A comparison of the level of hyperuniformity in all these active systems shows that strong self-avoidance steering of iABPs generates a similar level of weak - class III - hyperuniformity (with $\alpha \simeq 0.5$) as in the other active systems investigated so far. A related system of a binary mixture of programmable self-propelled robots, but with a different steering mechanisms of non-reciprocal motion alignment, has been shown to also exhibit weak hyperuniformity, with $\alpha = 0.45$, at the transition between ‘absorbing’ and ‘active‘ states. These two states correspond to particles trapped locally with suppressed density fluctuations, and particles with continuous rearrangement and diffusive dynamics^54^. The advantage of our system with self-steering avoidance is that a hyperuniform distribution of agents can be obtained in a single-component system, and also does not require any fine-tuning of parameters.

It would be interesting to extend this study in the future in several directions. For example, the intruder can have a larger speed than the agents, which may require a different optimal strategy for crowd penetration. Also, crowd penetration in three spatial dimensions should display novel features, because more empty space is available for avoiding collisions.

## Supplementary Information

Supplementary Information 1.

Supplementary Information 2.

Supplementary Information 3.

## Data Availability

Data supporting the results include intruder trajectories for various maneuverabilities, as well as time-dependent particle positions for agents in the crowd, are available in Zenodo. The data sets are accompanied by evaluation codes to extract intruder directional speed, mean squared displacement and transverse diffusivity, as well as hyperuniformity estimates for the agent crowd. The datasets generated and/or analysed during the current study are available in Zenodo, 10.5281/zenodo.18623049.
