# Supplementary material for: Directional motion of a self-steering active intruder in a dense crowd of cognitive active agents
Source: Sci Rep. 2026 Jun 15;16:18525. doi: 10.1038/s41598-026-52749-4 (PMC13269928; doi:10.1038/s41598-026-52749-4)
Supplement: Supplementary file 3 — Supplementary Information 3. [file 41598_2026_52749_MOESM3_ESM.pdf]

Directional motion of a self-steering active intruder in a dense crowd of cognitive active agents
— Supplementary Information —

Vikas Kumar Kushwaha,[1] Priyanka Iyer,[2] Sunil P. Singh,[1] and Gerhard Gompper[2,*]

[1]*Department of Physics, Indian Institute of Science Education and Research Bhopal, Bhopal, India*
[2]*Theoretical Physics of Living Matter, Institute for Advanced Simulation, Forschungszentrum Jülich, 52425 Jülich, Germany*

S1. INTRUDER'S MOTION OF INTRUDER WITH ONLY DIRECTIONAL MANEUVERABILITY

A. Orientational probability and auto-correlation function

The equation of motion for the orientation angle ψ_i of the intruder is

$$\dot{\phi}_i^I = -\frac{\Omega_v^I}{N_{c,i}} \sum_{j \in VC} e^{-r_{ij}/R_0} \sin(\psi_{ij} - \phi_i) - \Omega_d^I \sin\phi_i + \Lambda_i(t), \tag{S1}$$

When the vision maneuverability of the intruder is zero, $\Omega_v^I = 0$, i.e., the intruder has only directed maneuverability Ω_d^I, this equation simplifies to

$$d\phi(t)/dt = \sqrt{2D_r}\zeta(t) - \Omega_d^I \sin(\phi(t)) \tag{S2}$$

The equivalent Fokker-Planck equation is

$$\frac{\partial P(\phi,t)}{\partial t} = -\frac{\partial}{\partial \phi}(-\Omega_d^I \sin(\phi)\ P(\phi,t)) + \frac{1}{2}\frac{\partial^2}{\partial \phi^2}(2D_R\ P(\phi,t)) \tag{S3}$$

With the assumption of small variations of the angle ϕ around the goal direction, so that $\sin(\phi) \approx \phi$, the Fokker-Planck equation can be solved to find the time-evolving probability distribution of the process $\phi(t)$,

$$P(\phi,t) = \frac{1}{\sqrt{2\pi(D_R/\Omega_d^I)(1-\exp(-2\Omega_d^I t))}} \exp\left[-\frac{(\phi - \phi_0\ \exp(\Omega_d^I t)^2}{2(D_R/\Omega_d^I)(1-\exp(-2\Omega_d^I t)}\right]. \tag{S4}$$

As the derivation of $P(\phi,t)$ involves the approximation $\sin(\phi) \approx \phi$, Eq. (S4) and results based on it are valid for $\Omega_d^I > D_R$.

With the help of the probability density $P(\phi,t)$, we compute the orientational auto-correlation function

$$C(t) = \langle e^{i\phi(t)} \cdot e^{-i\phi(0)} \rangle \tag{S5}$$

For simplicity, we choose the reference angle $\phi(0) = 0$, so that $C(t) = \langle e^{i\phi(t)} \rangle$. This yields the explicit expression

$$C(t) = \int d\phi\, e^{i\phi} \frac{1}{\sqrt{2\pi(D_R/\Omega_d^I)(1-\exp(-2\Omega_d^I t))}} \exp\left[-\frac{(\phi - \phi_0\ \exp(\Omega_d^I t)^2}{2(D_R/\Omega_d^I)(1-\exp(-2\Omega_d^I t)}\right] \tag{S6}$$

and finally

$$\boxed{C(t) = \exp\left[-\frac{1}{2}\frac{D_R}{\Omega_d^I}(1 - e^{-2\Omega_d^I t})\right]} \tag{S7}$$

This result implies that the relaxation time τ for reaching the steady state is $\tau = (2\Omega_d^I)^{-1}$.

B. Mean square displacement and transverse diffusivity

With the help of Eq. (S7), we can also calculate the mean square displacement of the intruder perpendicular to the goal direction, which characterizes the deviations of the intruder's trajectory from a straight motion in the goal direction.

The instantaneous position of the intruder is

$$\mathbf{r}(t) = v_0 \int_o^t \hat{e}(t')dt'\ , \tag{S8}$$

so that the mean square displacement can be expressed as

$$\langle \mathbf{r}^2(t) \rangle = v_0^2 \int_o^t \int_o^t \langle \hat{e}(t') \cdot \hat{e}(t'') \rangle\ dt'dt''\ , \tag{S9}$$

which contains again the orientational auto-correlation function $C(|t-t'|) = \langle \hat{e}(t') \cdot \hat{e}(t'') \rangle$, compare Eq. (S5). Equation (S9) is solved by substituting Eq. (S7) to obtain

$$\langle \mathbf{r}^2(t) \rangle = v_0^2 \left(1 - \frac{D_R}{2\Omega_d^I}\right) t^2 + v_0^2 \frac{D_R}{2(\Omega_d^I)^2}\left(t + \frac{1}{2\Omega_d^I}(e^{-2\Omega_d^I t} - 1)\right)\ . \tag{S10}$$

The first term on the right-hand side represents the mean-square displacement in the goal direction, and the

* g.gompper@fz-juelich.de

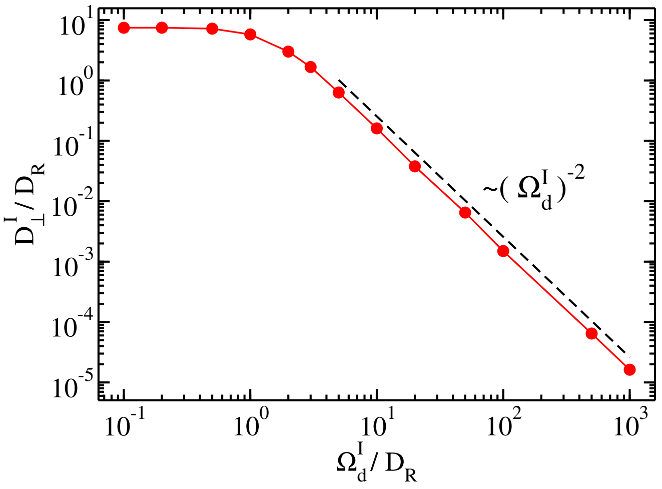

FIG. S1. The transverse diffusivity $D_{\perp}^{I}/D_R$ of the intruder as a function of directed maneuverability, when the intruder has activity $Pe_I = 4$ and vision maneuverability $\Omega_v^I = 0$.

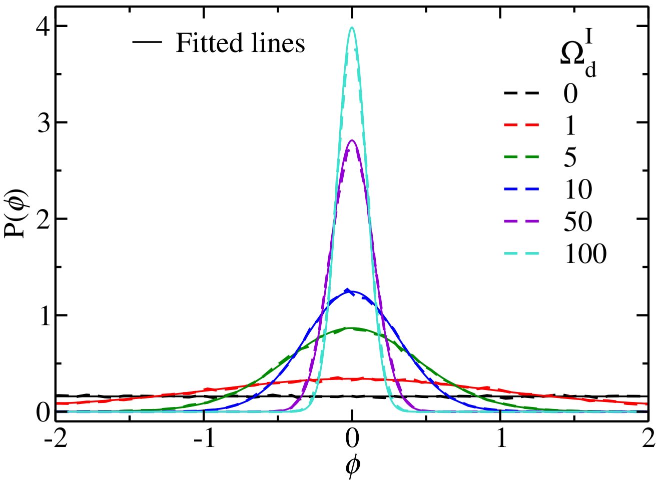

FIG. S2. Histogram of the intruder's orientation angle ϕ for the directed maneuverabilities $\Omega_d^I \in (0, 100)$. The dashed lines represent the simulation data, while the solid lines represent the theoretical distribution obtained from Eq. (S14), fitted to the simulation results for the corresponding values of Ω_d^I.

second term the corresponding displacement in the perpendicular (y) direction. Thus,

$$\langle \mathbf{y}^2(t)\rangle = v_0^2 \frac{D_R}{2(\Omega_d^I)^2}\left(t + \frac{1}{2\Omega_d^I}(e^{-2\Omega_d^I t} - 1)\right) \tag{S11}$$

The transverse diffusivity

$$D_{\perp}^{I} = \lim_{t\to\infty} \frac{1}{2}\frac{d}{dt}\langle \mathbf{y}^2(t)\rangle. \tag{S12}$$

can be obtained directly from Eq. (S11),

$$\boxed{D_{\perp,0}^{I} = \frac{v_0^2 D_R}{4(\Omega_d^I)^2}}, \tag{S13}$$

which is valid for $\Omega_d^I \gg D_R$. This result implies that in the absence of vision-based maneuverability of the intruder – or in the absence of agents – the transverse diffusivity has an inverse-square dependence on the intruder's directed maneuverability.

The comparison of the analytical result (S13) for the transverse diffusion of the intruder in the absence of agents is displayed in Fig. S1. The simulations show that the diffusivity remains approximately constant for small values of Ω_d^I/D_R, then decreases for $\Omega_d^I/D_R > 10$, following an inverse-square dependence. This behavior is in excellent agreement with the theoretical prediction.

C. Orientation distribution and averages

The stationary state solution of equation (S3), i.e for $\frac{\partial P(\phi,t)}{\partial \phi} = 0$, is given by

$$P(\phi) = \frac{\exp\left(\Omega_d^I \cos\phi/D_R\right)}{2\pi I_0(\Omega_d^I/D_R)}, \tag{S14}$$

where $I_n(...)$ denotes the modified Bessel function of the first kind.

We find that in the absence of agents, the probability distribution function of the orientation angle (ϕ) of the intruder has a peak at $\phi = 0$ (i.e., oriented towards the goal) and becomes narrow [1], in agreement with the prediction of Eq. (S14). Additionally, the average directed velocity satisfies the condition

$$\langle V_{\parallel}\rangle/v_0 = \langle \cos(\phi)\rangle. \tag{S15}$$

The average orientation of the intruder is denoted by $\langle\cos(\phi)\rangle$ and is given by the expression [2]

$$\langle\cos(\phi)\rangle = \int_0^{2\pi} d\phi\, P(\phi)\cos(\phi) = \frac{I_1(\Omega_d^I/D_R)}{I_0(\Omega_d^I/D_R)}. \tag{S16}$$

Figure S3 shows the comparison between simulation and theoretical results [1]. As the directed maneuverability Ω_d^I increases, the speed approaches the maximum value of $\langle V_{\parallel}\rangle/v_0 \simeq 1$ with the scaling

$$\langle V_{\parallel}\rangle/v_0 = 1 - \frac{D_R}{2\Omega_d^I}, \tag{S17}$$

in the limit of $\Omega_d^I/D_R \gg 1$.

We change the agent's vision maneuverability Ω_v^A/D_R, while keeping the intruder's parameters fixed at $\Omega_v^I = 0$, and all other properties the same as Tab. I of the main text, except $\Omega_d^I = 5$, 10, and 20. Figure S4 shows that there is a significant effect of the agent's vision maneuverability on the intruder's directional motion, as with increasing Ω_v^A/D_R, agents can move out of the way of the intruder and thereby reduce the frequency of direct collision. Thus, the intruder speed $\langle V_{\parallel}\rangle$ increases with Ω_v^A/D_R.

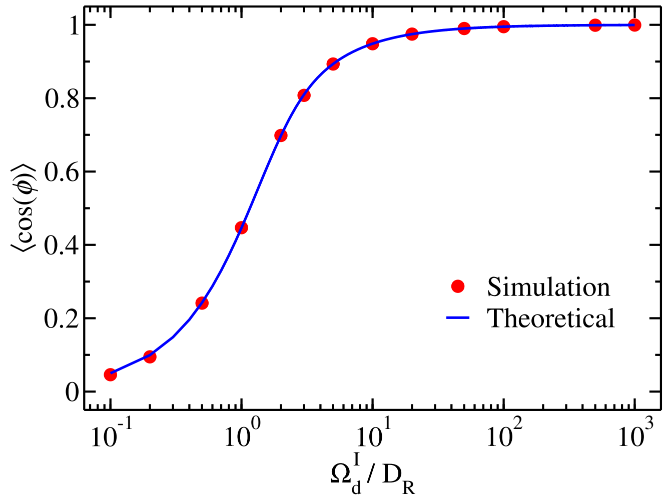

FIG. S3. Average orientation $\langle\cos(\phi)\rangle$ of the intruder relative to the goal direction $\mathbf{e}_g$, as a function of directed maneuverability Ω_d^I/D_R, with simulation data (red bullets) and theoretical results (solid black line) obtained from Eq. (S16).

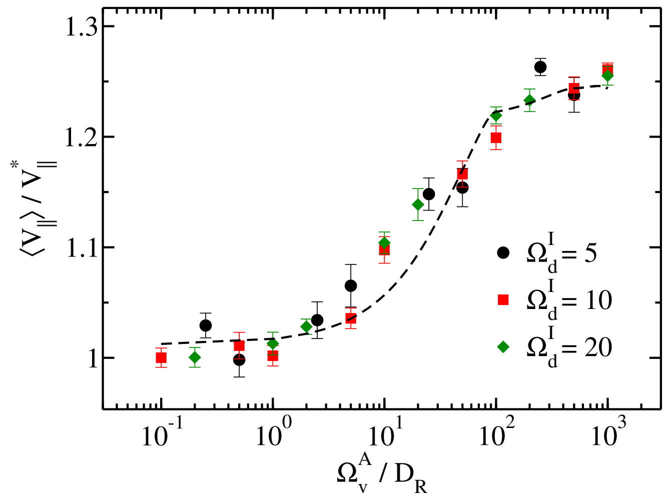

FIG. S4. The variation of the intruder's normalized directed velocity for zero intruder maneuverability ($\Omega_v^I = 0$) as a function of the agents' maneuverability Ω_v^A/D_R, shown for different values of the intruder's directed maneuverability $\Omega_d^I = 5, 10$, and 20.

S2. MOTION OF AN INTRUDER WITH VISION-INDUCED MANEUVERABILITY

A. Relation of transverse diffusivity and directional speed – a toy model

A comparison of Figs. 2(a) and 6(a) of the main text suggests that there is a strong (inverse) relation between the directional speed $\langle V_\parallel\rangle$ and the transverse diffusivity $D_\perp^I$ of the intruder.

In order to comprehend this relation, we consider a simplified toy model, where particles are assumed to move on a square lattice with lattice constant a. Particles move along the bonds from vertex to vertex in time steps Δt. Without vision-guided avoidance, particles move unobstructed with speed $v_0 = a/\Delta t$. Scattering events due to avoidance steering reduce the probability for a particle to reach the next vertex in the goal direction, and simultaneously increase the probability to move in the transverse direction. Let p be the probability to move in the goal direction, and $(1-p)$ to move in the transverse direction.

This implies for the average directional speed that $\langle V_\parallel\rangle = pa/(\Delta t) = pv_0$, so that $p = \langle V_\parallel\rangle/v_0$. For the transverse diffusivity, the model yields $D_\perp^I = (1-p)^2a^2/(\Delta t)$. Then, with the assumption that the typical time scale is proportional to the inverse rotational diffusion coefficient $1/D_R$, and $p = \langle V_\parallel\rangle/v_0$, we finally obtain

$$D_\perp = D_T^{(ABP)}(1-\langle V_\parallel\rangle/v_0)^2, \qquad \text{(S18)}$$

where $D_T^{(ABP)}$ is the translational diffusion coefficient of an active Brownian particle.

The important factor is $(1-\langle V_\parallel\rangle/v_0)^2$, because $\langle V_\parallel\rangle/v_0 = \Gamma(\Omega_v^I/\Omega_d^I)$, with scaling function $\Gamma(.)$, compare Eq. (11) of the main text. Thus, Eq. (S18) makes plausible that in the presence of vision-induced avoidance steering

- the functional dependence of $D_\perp^I$ on Ω_v^I/Ω_d^I
- the "inverted" dependence of $\langle V_\parallel\rangle/v_0$ and $D_\perp$, i.e. $x\langle V_\parallel\rangle/v_0$ decreases as a function of Ω_v^I/Ω_d^I, while $D_\perp$ increases
- the transition from goal-direction dominated to visual-avoidance dominated behavior occurs in both cases at $\Omega_v^I/\Omega_d^I \simeq 10$
- and is consistent with Eq. (S13) in the limit $\Omega_v^I \to 0$ and $\Omega_d \gg D_r$ (using Eq. S17).

Thus, this simplified toy model explains the main qualitative features of $D_\perp$.

B. Effect of vision-induced steering attraction of the intruder by agents

If we change the sign of the vision maneuverability of the intruder $\Omega_v^I \to -\Omega_v^I$, then instead of avoiding the agents, the intruder follows them. In such a case, the average directed velocity of the intruder again decays with the magnitude of the maneuverability ratio, see Fig. S5. Somewhat surprisingly, the velocity exhibits an approximately symmetric behavior at high density, i.e. $\langle V_\parallel(-\Omega_v^I)\rangle \simeq \langle V_\parallel(\Omega_v^I)\rangle$. This can be understood intuitively as follows: At high agent density, the agent distribution is nearly isotropic but shows some fluctuations. Then, it does not matter much for the distraction of the intruder from the goal direction whether it reorients more toward the somewhat more dense or the somewhat less dense regions.

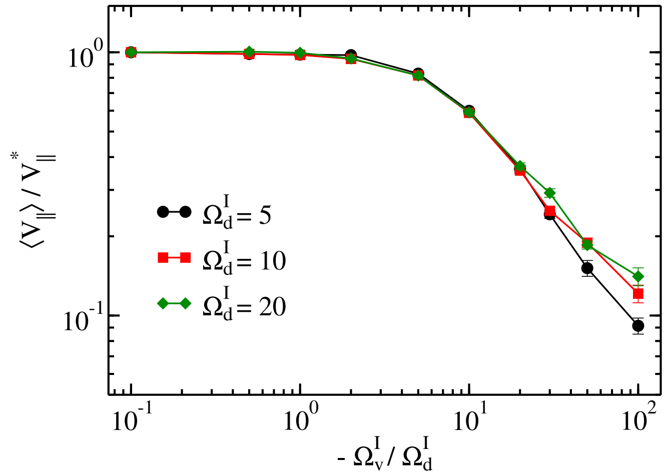

FIG. S5. The variation of the intruder's normalized directed velocity as a function of the vision maneuverability at three different directed maneuverabilities when $\Omega_v^I \to -\Omega_v^I$.

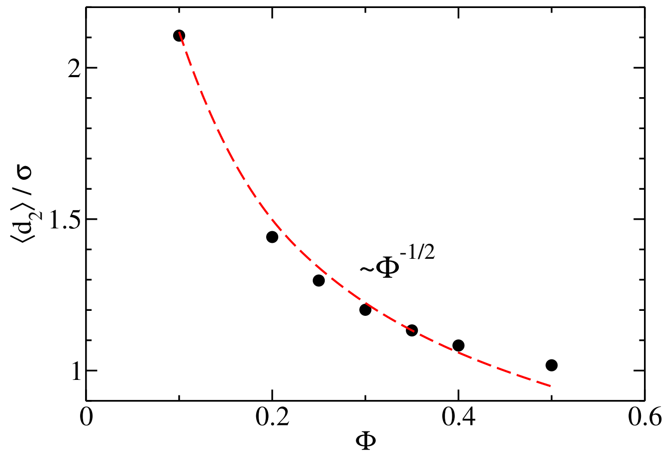

FIG. S6. The average distance between agents ($< d_2 > /\sigma$) as a function of area packing fraction.

S3. AVERAGE DISTANCE BETWEEN AGENTS

The average inter-agent distance is independent of intruder–agent interactions, since it is an intrinsic property of the agents. Figure S6 shows the dependence of average inter-agent distance $\langle d_2 \rangle/\sigma$ as a function of packing fraction Φ, following the scaling $\langle d_2 \rangle = a_0 \Phi^{-1/2}$ with a fitted prefactor $a_0 = 0.67$. The numerical result of the prefactor (a_0) can be compared with the lattice constant of a regular triangular lattice as a reference. The area fraction of the system is

$$\Phi = \frac{N\pi\sigma^2}{4L^2}. \tag{S19}$$

For a triangular lattice, the number of particles in a triangle formed by three particles is $n = 3 * 60/360 = 1/2$, and the area of a triangle is $A_t = \sqrt{3}d_t^2/2$, where d_t is the length of the triangle side. Therefore, the total occupied area $A = NnA_t = N\sqrt{3}d_t^2/4$, which equals the area of the simulation box, i.e., $A = L^2$. Hence, we obtain $L^2 = \sqrt{3}Nd_t^2/4$, or $d_t^2 = 4L^2/(\sqrt{3}N)$. By multiplying with equation with Eq. (S19), we get

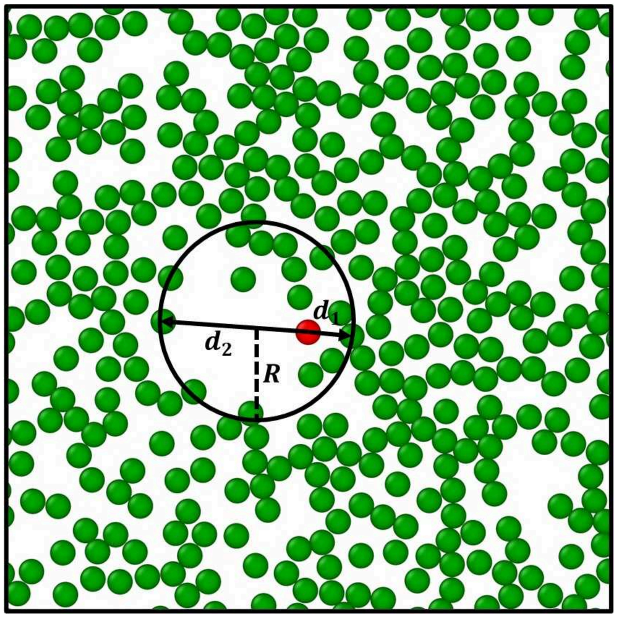

FIG. S7. Typical agent distribution around the intruder, with geometric parameters of the void region. Here, d_1 is the distance between the intruder and the nearby front agent and d_2 the distance between the intruder and the nearby rear agent.

$$d_t = \sqrt{\frac{\pi\sigma^2}{\sqrt{3}}}\Phi^{-1/2} \tag{S20}$$

which provides the prefactor

$$a_0 = \sigma\pi^{1/2}3^{-1/4} \approx 0.67 \tag{S21}$$

Hence, the theoretically estimated value a_0/σ for the triangular lattice of the particles is in good agreement with the average minimal distance in a fluid of active agents obtained from simulations. This indicates that self-steering agents in this active fluid self-organize such as to keep nearly equal distances from their neighbors. This result is in good agreement with particles with avoidance steering in Ref. [3].

S4. AGENT DEPLETION AROUND INTRUDER

We calculate the radius of the region around the intruder, which is essentially void of agents, as shown in Fig. S7.

To quantify the size of this void, we first identify the nearest-neighbor agents within two angular sectors – in cones in front and behind the intruder – each with a narrow opening angle of 2Θ, where $\Theta = \pi/6$. These

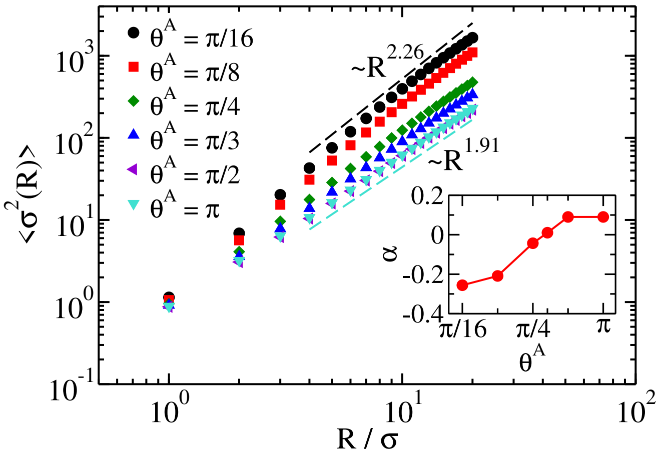

FIG. S8. Dependence of number variance $\langle\sigma_N^2(R)\rangle$ on the radius R of the observation window, for multiple agent vision angles $\theta^A \in (\pi/16, \pi)$ at a fixed agent maneuverability $\Omega_v^A = 16$. The inset shows the variation of the hyperuniformity parameter α as a function of θ^A.

front and rear distances are defined as

$$r_{\text{front}} = \min_i\{r_i \big| |\Delta\phi_i| \le \Theta\},$$
$$r_{\text{rear}} = \min_i\{r_i \big| |\Delta\phi_i - \pi| \le \Theta\}, \qquad \text{(S22)}$$

respectively, where $\Delta\phi_i = \phi_i - \psi_i$ is the relative angular position of the ith neighbor with respect to the intruder's orientation. The nearest agents within these domains define the front and rear neighboring distances, which together provide an estimate of the void's diameter. The mean void radius is then given by $R_{void} = [\langle r_{\text{front}}\rangle + \langle r_{\text{rear}}\rangle]/2$.

S5. HYPERUNIFORMITY

A. Effect of agent's vision angle

Hyperuniformity depends on the agent's parameters, such as vision angle, maneuverability, and activity.

The intruder's directional speed increases with the agents' field of view, as shown in the main text, Fig. 2(d). Hyperuniformity is related to this behavior, as the system becomes more hyperuniform with increasing vision angle θ^A, see Fig. S8. When the agents have a narrow field of view, their dynamics resemble those of simple active Brownian particles (ABPs). Collisions occur frequently, which slows their motion and induces the formation of small clusters. In contrast, a wide field of view allows agents to better anticipate and maneuver around one another, actively maintaining separation. As a result, the system evolves toward a more uniform spatial distribution. Notably, for $\theta^A \gtrsim \pi/2$, each agent effectively responds to all neighboring agents, and the system attains its maximum degree of uniformity, as shown in the inset of Fig. S8.

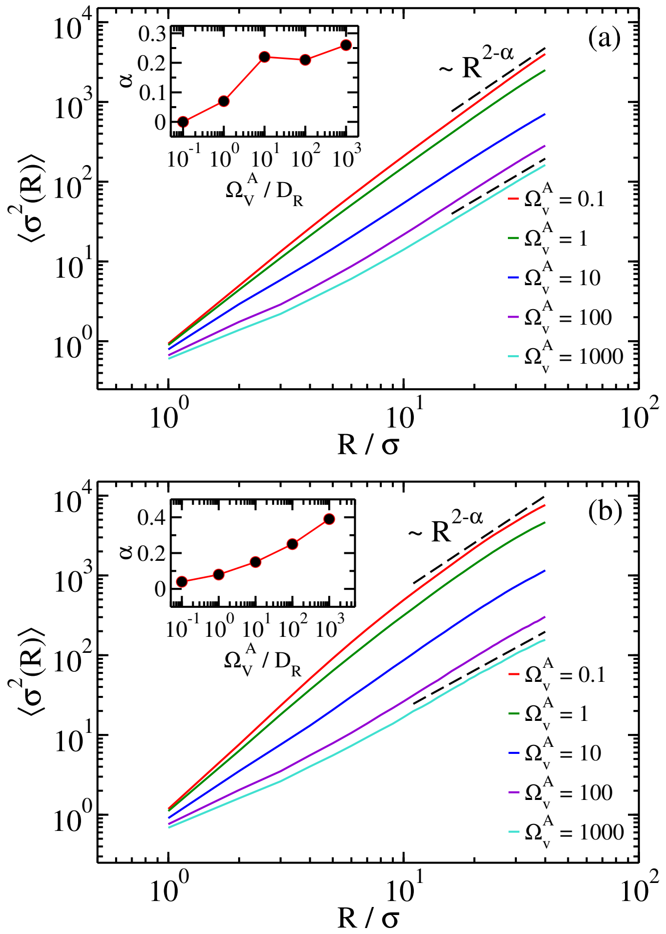

FIG. S9. Dependence of the number variance $\langle\sigma_N^2(R)\rangle$ of the agents' density distribution for various Ω_v^A, as indicated, for a larger range of radii R of the observation window – compare Fig. 7(b) of the main text – with packing fraction (a) $\Phi = 0.3$ and (b) $\Phi = 0.5$. The insets show the resulting quasi-asymptotic values of the hyperuniformity exponent α, which indicates a nearly random (Poisson-like) density distribution for small Ω_v^A, and a weak hyperuniformity for large Ω_v^A.

B. Finite-size effects and asymptotic behavior

Figure 7(b) of the main text displays the dependence of the number variance $\langle\sigma_N^2(R)\rangle$ of the agents' density distribution for a range $5 \le R/\sigma \le 25$ of radii R of the observation window. While this range seems relevant for comparison with the intruder directional motility, is does not allow the estimation of the asymptotic bahavior of $\langle\sigma_N^2(R)\rangle$ for large R, and therefore of the large-scale uniformity of the cognitive active fluid of agents. Therefore, we present in Fig. S9 results of $\langle\sigma_N^2(R)\rangle$ for larger R, obtained from additional simulations for system size $L = 100$ with packing fractions $\Phi = 0.3$ and $\Phi = 0.5$, together with the extracted values of the hyperuniformity index α. These results show that for for small Ω_v^A, where particles interact weakly and the system behaves almost like an ideal gas, the spatial organization displays

asymptotically (for large R) a Poisson-like random distribution with $\alpha \simeq 0$. For large Ω_v^A, the effective values of α for the different R-ranges in Figs. 7(b) and S9 do not differ much, with $\alpha \simeq 0.4$ indicating weak hyperuniformity. Furthermore, the comparison of the two packing fractions $\Phi = 0.3$ and $\Phi = 0.5$ in Figs. S9(a) and (b), respectively. indicates that the hyperuniformity depends only weakly on packing fraction, where α slowly increases with increasing Φ.

S6. MOVIE CAPTIONS

Movie M1: Dynamics of an intruder moving through a dense crowd of agents with maneuverability $\Omega_v^A = 16$, at $Pe^A = Pe^I = 4$, $\theta^A = \pi/2$, $\theta^I = \pi/4$, and $\Phi = 0.5$. The three panels of the movie correspond to different intruder maneuverability ratios, $\Omega_v^I/\Omega_d^I = 1$, 10, and 100, shown in the top, middle, and bottom panels, respectively. The evolving intruder trajectories are shown by red lines.

Movie M2: Dynamics of an intruder moving through a dense crowd of agents with enhanced maneuverability $\Omega_v^{A,I}$ with respect to the intruder. Other parameters are the agent-agent maneuverability $\Omega_v^A = 16$, directed maneuverability $\Omega_d^I = 10$, $Pe^A = Pe^I = 4$, $\theta^A = \pi/2$, $\theta^I = \pi/4$, and $\Phi = 0.5$. The three panels of the movie correspond to different agents maneuverability ratios near the intruder, $\Omega_v^{A,I}/\Omega_v^A = 0.625$, 6.25, and 31.25, shown in the top, middle, and bottom panels, respectively. The evolving intruder trajectories are shown by red lines.

[1] S. Othman, J. Midya, T. Auth, and G. Gompper, Phys. Rev. E **111**, 015425 (2025).
[2] S. Goh, R. G. Winkler, and G. Gompper, New J. Phys. **24**, 093039 [1 (2022).
[3] R. S. Negi, P. Iyer, and G. Gompper, Sci. Rep. **14**, 9443 (2024).
